# Supplementary material for: Countering reproducibility issues in mathematical models with software engineering techniques: A case study using a one-dimensional mathematical model of the atrioventricular node
Source: PLoS One. 2021 Jul 19;16(7):e0254749. doi: 10.1371/journal.pone.0254749 (PMC8289093; doi:10.1371/journal.pone.0254749)
Supplement: Attachment — Submitted filename: 2020-InaMo-rebuttal-rev1_final.pdf [file pone.0254749.s002.pdf]

Author Response to Reviews of

Countering reproducibility issues in mathematical models with software engineering techniques: A case study using a one-dimensional mathematical model of the atrioventricular node

Christopher Schölzel, Valeria Blesius, Gernot Ernst, Alexander Goesmann, and Andreas Dominik

PLOS ONE

June 10, 2021

Dear Dr. Bannister,

we thank you very much for the opportunity to revise our manuscript. We also thank the anonymous reviewer for their valuable comments. This revision incorporates the suggested improvements as well as some required changes that we noticed ourselves. The most important changes are listed in the following.

- As suggested by reviewer #1, we added a paragraph discussing interoperability and the related advantages of SBML/CellML in the discussion.
- We searched through the whole article for statements that painted the work of Inada et al. in an overly negative light and corrected them, eliminating judgmental language and highlighting the overall importance of the Inada model. We especially tried to clarify that we believe the source of the reproducibility issues is the complexity of the model and not a lack of diligence of the authors or the reviewers.
- Since InaMo 1.4.3, we now include an export of the main model as Functional Mock-up Unit (FMU), which should increase the interoperability of InaMo at least within the Modelica domain.
- We have switched from Travis CI to GitHub actions, because Travis CI is no longer free for public open source repositories. We therefore replaced all mentions of Travis CI as well as the code examples for the CI/CD scripts.
- Some statements in the manuscript did not reflect the fact that we performed a manual reconstruction of the data of *all* reference plots shown in the supplement. We updated the affected sentences accordingly.
- While going through the article, we found a few issues with spelling, grammar, and references. In particular, we updated the date on all web resources from 2020 to 2021, replaced bioRxiv references with published versions, and replaced the reference to Juty 2013 with Courtot 2011, because we believe that this is a better reference for the SBO.

Our detailed responses are listed in the following along with the original comments.

Kind Regards,
Christopher Schölzel, Valeria Blesius, Gernot Ernst, Alexander Goesmann, and Andreas Dominik

---

Reviewer #1

Comment 1.1

"My only criticism of the study would be that the authors' use of Modelica comes at the expense of more exchangeable formats such as CellML/SBML. The Modelica code provided with this submission is very complete, but the CellML and SBML formats are quite a bit more portable, and can be imported and exported by many tools, and the current study does not appear to have an accompanying CellML/SBML model. The authors argue that CellML/SBML unsuited to first-hand model authoring due to their lack of human readability and problematic interaction with version control systems. For this particular study, I agree with this assessment. I believe that providing CellML/SBML code for this model would be against the DRY principle and create synchronization issues and hence barriers to robust reproducibility (and certainly, it would not be difficult to derive a CellML/SBML version of this model given the wealth of tests and documentation provided by the authors). However, I would view it as a regression if exchangeable standards such as CellML/SBML were eventually replaced with less transferable counterparts. I think the authors can address these issues as discussion items, rather than make any implementation changes. I think an ideal solution would be to pursue an authoring medium, perhaps a meta-language, that retains the clarity and pragmatism of Modelica (for example) but can be interconverted to/from standards like CellML/SBML. Perhaps the authors can create a new paragraph in the discussion section that acknowledges the model exchangeability issue, considers the pros and cons of various approaches, and makes recommendations regarding how the field could approach a solution to this issue in the future. The authors already mention some of these issues around line 900. This section could be further expanded to offer recommendations and potential solutions for practical modeling languages and workflows that (I hope) attain all of the authors' criteria as well as exchangeability and annotation support that standards were designed to address."

Response: We fully agree with the reviewer, and thank them for pointing out this oversight. While we do favor Modelica for model design, CellML/SBML have an undeniable advantage as exchange formats. In fact, we would be happy to provide a separate SBML or CellML version of the model, if there was a way to generate it automatically from Modelica code. As far as we are aware, such a conversion is currently only possible in the opposite direction with SBML2Modelica. This provides some interoperability between the two domains, but is insufficient to make our implementation directly accessible within an SBML- or CellML-based tool. The following approaches could remedy this in future projects:

- SBML/CellML tools could be extended to support the Functional Mock-up Interface (FMI), which is the designated exchange format in the Modelica domain. The only modeling tool we are aware of that supports both FMI and SBML is MATLAB with the SimBiology and FMI toolboxes. To facilitate this form of interoperability, we now also provide the three main model as Functional Mock-up Unit (FMU). The downside is that FMUs are opaque boxes, which do not allow a partial reproduction of results that requires a structural modification of the model.

- Like the reviewer said, a standard, human-readable text representation of CellML/SBML models with sufficient support for modularity would allow to apply the same techniques presented in this article to CellML/SBML models. Antimony and CellML Text come to mind as possible candidates.

- A full automatic translation of Modelica to SBML/CellML would probably be impossible, because SBML/CellML does not have full support for DAEs, discrete variables, and the object-oriented modeling style of Modelica. However, it could be achieved for a well-defined subset of the language. Upon compilation, Modelica models are "flattened", removing the hierarchical structure and collecting all variables and equations in one single file. Starting from this representation, a translation to SBML/CellML seems achievable, yet still quite complicated due to discrete events, Kirchhoff-like algebraic equations, and custom functions with imperative syntax. As an additional downside, the "flat" Modelica code loses most of the benefits regarding understandability and modularity of the model.

As suggested by the reviewer, we added a paragraph in section 4.3 that discusses this issue.

Editor

Comment 2.1

"My impression of the manuscript is that it is thorough, meticulously-researched and of use to the field. However, the many expressions of frustration with the Inada model should be minimized throughout the text."

Response: We agree with the editor. We already tried to avoid emotional or judgmental language, but realized that we can do a better job to keep a factual tone throughout the text. In particular, we noticed that the reader can get the

impression that we blame the authors and/or the reviewers of the Inada et al. study for the reproducibility issues. However, while we cannot deny that we felt frustration throughout the development, we believe that such issues are a natural result of high model complexity and that they can only be solved reliably with automated tool support. We tried to clarify this throughout the manuscript and also highlight that we do believe that Inada et al. were interested in making their methods as transparent as possible. In a way this makes the Inada model even more interesting as an example, because it shows that even high quality research is subject to the issues that we encountered.
